# Nutritional performance and activity of some digestive enzymes of the cotton bollworm, *Helicoverpa armigera* , in response to seven tested bean cultivars

**DOI:** 10.1093/jis/14.1.93

**Published:** 2014-07-17

**Authors:** Foroogh Rahimi Namin, Bahram Naseri, Jabraeil Razmjou, Allen Cohen

**Affiliations:** Department of Plant Protection, Faculty of Agriculture, University of Mohaghegh Ardabili, Ardabil, Iran

**Keywords:** amylolytic activity, nutritional responses, proteolytic activity

## Abstract

Nutritional performance and activity of some digestive enzymes (protease and α -amylase) of *Helicoverpa armigera* Hübner (Lepidoptera: Noctuidae) in response to feeding on bean ( *Phaseolus vulgaris* L. (Fabales: Fabaceae)) cultivars (Shokufa, Akhtar, Sayyad, Naz, Pak, Daneshkadeh, and Talash) were evaluated under laboratory conditions (25 ± 1°C, 65 ± 5% RH, and a 16:8 L:D photoperiod). The highest and lowest respective values of approximate digestibility were observed when fourth, fifth, and sixth larval instar *H. armigera* were fed red kidney bean Akhtar and white kidney bean Daneshkadeh. The efficiency of conversion of ingested and digested food was highest when *H. armigera* was fed red kidney beans Akhtar and Naz and lowest when they were fed white kidney bean Pak. The highest protease activity of fifth instars was observed when they were fed red kidney bean Naz, and the highest amylase activity of fifth instars was observed when they were fed red kidney bean Sayyad. Sixth instar larvae that fed on red kidney bean Sayyad showed the highest protease activity. Larvae reared on common bean Talash and white kidney bean Pak showed the highest amylase activity. Among bean cultivars tested, red kidney bean Sayyad was the most unsuitable host for feeding *H. armigera* .

## Introduction

Nutrition is known as the chemicals an organism requires for its growth, tissue maintenance, reproduction, and energy supply. Although insects can synthesize some of these chemicals, they acquire the majority of them by feeding ( Chapman 1998 ). Survival, development, and reproduction of phytophagous insects are considerably affected by the primary and secondary chemical compositions of host plants; hence, food consumption and utilization depend on both plant quality and insect nutritional performance ( Scriber and Slansky 1981 ; Singh and Mullick 1997 ). Food quality affects rate of development, body composition, and growth of arthropods considerably ( Waldbauer 1968 ). Waring and Cobb (1992) reported that physiological and biochemical changes, including nutritional and allelochemical changes, in a plant can alter its nutritional value for herbivores. Therefore, much effort has been focused on nutritional physiology, studying the effects of nutritive compounds and secondary metabolites on insect responses, such as preingestive increases in consumption of nutritionally-poor food or postingestive increases in activity of digestive enzymes ( Mattson 1980 ; Taylor 1989 ; Duffy and Stout 1996; Lazarevic 2000 ).

Like other insect orders, the balance of nutrients in many lepidopterans is important ( Genc 2006 ). Lepidopteran insects respond to unsuitable diets in diverse ways, such as altering the amount of ingested food, switching from one food source to another, and/or regulating the efficiency of the nutrients ( Genc 2006 ). They typically require proteins, amino acids, and carbohydrates in approximately equal amounts ( Chapman 1998 ; Nation 2001 ).

The cotton bollworm, *Helicoverpa armigera* Hübner (Lepidoptera: Noctuidae), a polyphagous insect pest that attacks most plant structures (including stems, leaves, flower heads, and fruits; Moral Garcia 2006 ), feeds on economically important bean crops in Iran and elsewhere in the world ( Farid 1986 ; Reddy et al. 2004 ). In spite of a high level of natural mortality, *H. armigera* needs to be controlled by chemical methods in order to prevent large agricultural losses ( Fitt 1994 ). However, environmental concerns and the development of pesticide resistance, especially to pyrethroids ( Gunning et al. 1984 ), has generated considerable interest in other strategies for *H. armigera* management ( Naseri et al. 2009 ).

Plants with antibiosis defenses have negative effects on insect survival, size, weight, longevity, and reproduction. Host plant resistance is an acceptable and useful method economically and has been used effectively in pest management strategies for several insect pests ( Kennedy et al. 1987 ; Dent 2000 ; Sarfraz et al. 2006 ). Because the digestive tract is the key interface between insects and the environment, selective inhibition of digestive enzymes in many insect pests can be one of the most important mitigating effects of pest control strategies ( Bigham and Hosseininaveh 2010 ).

In this study, nutritional indices and digestive enzyme (α-amylase and protease) activity were determined in order to assess the effects of feeding seven diverse bean ( *Phaseolus vulgaris* L. (Fabales: Fabaceae)) cultivars to *H. armigera* . Because the tested bean cultivars have varying nutritional value for *H. armigera* larvae, it was hypothesized that they would accumulate biomass more efficiently when fed some bean cultivars rather than others, and that larvae raised on cultivars with lower protein and starch contents would have lower digestive enzyme activity, resulting in decreased efficiency of conversion of ingested and digested food into body matter.

Many previous studies have investigated insect responses to different host plant diets. Cohen and Patana (1984) evaluated the efficiency of food utilization by *Heliothis zea* (Boddie) fed either artificial diets or green beans. Several authors in the fields of nutritional and digestive physiology have recently evaluated the effects of different host plants on *H. armigera* ( Kotkar et al. 2009 ; Fallahne-jad-Mojarrad et al. 2010, 2011; Naseri et al. 2010a , b; Soleimannejad et al. 2010 ; Hemati et al. 2012a , b). Baghery et al. (2011) studied the effects on nutritional indices of *H. armigera* when incorporating different host plants, including corn, neavybean, cowpea, chickpea, and soybean, into artificial diets. The effects of different corn hybrids on nutritional responses of *H. armigera* larvae were investigated by Arghand et al. (2011) . There are no comprehensive physiological studies of *H. armigera* fed various bean cultivars, and the cultivars examined in this study are different from those tested by other researchers ( Cohen and Patana 1984 ; Hemati et al. 2012a , b). The results of this research will provide useful information for designing comprehensive pest management strategies against the cotton bollworm.

## Materials and Methods

### Plant sources

Seeds of seven different bean ( *P. vulgaris)* cultivars, including white kidney bean (cultivars Daneshkadeh, Pak, and Shokufa), red kidney bean (cultivars Akhtar, Naz, and Sayyad), and common bean (cultivar Talash), were acquired from the Plant and Seed Modification Research Institute (Khomein, Iran). They were grown in the research field of the University of Mohaghegh Ardabili (Ardabil, Iran) in May 2011. For this study, the young leaves and green terminal pods (all of the same size) of various bean cultivars were transferred to a growth chamber at 25 ± 1°C, 65 ± 5% RH, and with a 16:8 L:D photoperiod when the cultivars reached the reproductive stage. Experiments were conducted between morning and afternoon from mid-July to mid-September 2011. The leaves of bean cultivars were used to feed first and second *H. armigera* larval instars, and the green pods were used to feed third to sixth *H. armigera* larval instars ( Green et al. 2002 ; Naseri et al. 2009 ).

### Insect rearing

*H. armigera* eggs were acquired from a laboratory colony maintained on a cowpea-based artificial diet, as described by Teakle (1991) , from Tarbiat Modares University, Iran. The insects had already been reared for two generations on the same cultivars they were fed during experiments. All experimental insects were kept inside a growth chamber at 25 ± 1°C, 65 ± 5% RH, and with a 16:8 L:D photoperiod.

### Chemicals

All enzyme substrates, the Bradford reagent, and the dinitrosalicylic acid (DNS) were obtained from Sigma Chemical Co. (Sigma-Aldrich, www.sigmaaldrich.com ). Bovine serum albumin (BSA) and potassium iodine (KI) were respectively purchased from Roche Co. (Roche, www.roche.com ) and Merck Co. (Merck, www.merck.com ). Iodine (I2 **)** was obtained from Maarssen Co.

### Nutritional indices experiment

Fifty newly hatched larvae of *H. armigera* were transferred into plastic containers (diameter 16.5 cm, depth 7.5 cm), with outlets covered by a mesh net for larval aeration, containing the fresh leaves of each test cultivar. The petioles of detached leaves were inserted in water-soaked cotton to preserve freshness. Nutritional indices were quantified using fourth to sixth instar larvae because they were more measurable than the younger instars. The first and second instar larvae were simultaneously reared until the third instar, after which they were divided into individual plastic plates (diameter 8 cm, depth 1 cm) to prevent cannibalism ( Twine 1971 ). For prepupation and pupation, sixth instar larvae were kept in small plastic tubes (diameter 2 cm, depth 5 cm). Nutritional indices (including weight gain, food utilization, and feces produced by the larvae) were calculated grav-imetrically following Waldbauer (1968) . Fourth instar larvae were set up on the pods of bean cultivars after weighing. The weights of the larvae were recorded daily before and after feeding until feeding stopped and the prepupal stage was reached. The provided fresh foods and the remaining food and feces at the end of each experiment were weighed daily. The quantity of food ingested was calculated by subtracting the weight of remaining food at the end of each experiment from the weight of fresh food supplied. All above-mentioned weights were recorded as percentage of dry weights. The remaining food and produced feces of 20 specimens of newly molted larvae (fourth, fifth, and sixth instars) were collected, weighed, dried at 60°C for 48 hours, then weighed again.

Nutritional indices were calculated via formulae described by Waldbauer (1968) ; CI = consumption index, AD = assimilation efficiency, also called approximate digestibility, ECI = efficiency of conversion of ingested food, ECD = efficiency of conversion of digested food, RCR = relative consumption rate, and RGR =relative growth rate.

$CI=\frac{E}{A}$

$AD=\frac{E-F}{E}$

$ECI=\frac{P}{E}$

$ECD=\frac{P}{E-F}$

$RCR=\frac{E}{A^*T}$

$RGR=\frac{P}{A^*T}$

where, A = mean dry weight of insect per unit time, E = dry weight of food consumed, F = dry weight of feces produced, P = dry weight gain of insect, and T = duration of feeding period.

### Larval mortality and growth index

Percentage mortality of larvae, pupal weight, larval growth index (LGI), and standardized insect-growth index (SII) of *H. armigera* were calculated on different bean cultivars (Pretorius 1976; Itoyama et al. 1999 ):

$LGI=l_x/L$

$SII=P_w/L$

where, l _x_ = survival rate of larvae, L = larval period, and P _w_ = pupal weight.

### Preparation of digestive enzymes

Larval rearing methods for this part of the study were similar to those mentioned in the “Insect Rearing’ section. The fifth and sixth instar larvae of *H. armigera* that fed on pods of bean cultivars were anesthetized on ice and immediately dissected under a stereoscopic microscope. The midguts were washed in pre-cooled distilled water and unwanted tissues were removed. They were then submerged in a known volume of distilled water. The homogenates were centrifuged at 16,000 × g for 10 min at 4°C, and the resulting supernatants were collected in new micro tubes and stored at -20°C in aliquots for further use (Hosseininaveh et al. 2007).

### Protein quantification of larvae

General protein concentrations in the midgut of fifth and sixth instar larvae of *H. armigera* were determined using BSA as a standard according to Bradford (1976) .

### Proteolytic activity assay

General protease activity in the midgut of *H. armigera* larvae that fed on various bean cultivars for 24 hr was determined using azocasein substrate at optimal pH. The universal buffer system (50 mM sodium phosphate-borate) was used to assay the optimal pH of proteolytic activity in the midgut ( Elpidina et al. 2001 ). To evaluate the azocaseinolytic activity, the reaction mixture containing 80 µL of 1.5% azocasein solution in 50 mM universal buffer (pH 12) and 50 µL of crude enzyme was incubated at 37°C for 50 min. The reaction was terminated by the addition of 100 µL 30% trichloroacetic acid (TCA), followed by cooling at 4°C for 30 min and centrifugation at 16,000 × g for 10 min. The supernatant (100 µL) was added to 100 µL of 2 M NaOH, and the absorbance was read at 440 nm. Appropriate blanks, to which TCA had been added prior to the substrate, were prepared for each treatment. One unit of protease activity was defined as an increase in optical density per mg tissue protein per min due to azocasein proteolysis ( Elpidina et al. 2001 ). All experiments were carried out in triplicate (with three different supernatants).

### Amylolytic activity assay

The DNS method ( Bernfeld 1955 ), with 1% soluble starch at optimal pH as substrate, was used to assay the digestive amylolytic activity of *H. armigera* larvae that fed on different bean cultivars for 24 hr. A quantity of 20 µL of the enzyme extract was incubated with 500 µL of universal buffer (pH 10) and 40 µL of soluble starch for 30 min at 37°C. The reaction was stopped by addition of 100 µL DNS and heating in boiling water for 10 min. The absorbance was read at 540 nm after cooling on ice. Unit activity was characterized as the amount of enzyme required to produce 1 mg of maltose in 30 min at 37 °C under the given assay conditions. A standard curve of α -amylase absorbance against the amount of maltose released was constructed to facilitate the calculation of the amount of maltose released during the α -amylase assays. Serial dilutions of maltose in the universal buffer (pH 10) were made to produce the following concentrations: 0.125, 0.25, 0.5, 1, and 2 mg mL ^-1^ . The reaction mixture containing 50 µL of soluble maltose, 270 µL distilled water, and 50 µL of DNS was heated in boiling water for 10 min, and then the absorbance was read at the above-mentioned wave length. All experiments were carried out in triplicate (with three different supernatants).

### Protein and starch determination of bean cultivars given to larvae

Protein content of bean cultivars was quantified via BSA as standard according to Bradford (1976) . A quantity of 200 mg of each bean cultivar was homogenized in 10 mL of distilled water. 100 µL of the homogenate was then added to 3 mL of Bradford reagent. The samples were incubated in darkness at 37°C and absorbance was read at 595 nm.

Starch content of bean cultivars was determined by the method of Bernfeld (1955) using starch as standard. A quantity of 200 mg of each bean cultivar was homogenized in 35 mL of distilled water and heated to boiling point. 100 mL of each sample was added to 2.5 mL of iodine reagent (0.02% I _2_ and 0.2% KI) and absorbance was read at 580 nm.

### Data analysis

Nutritional indices and digestive enzyme activity of *H. armigera* reared on various bean cultivars were analyzed with one-way ANOVA followed by comparison of the means with LSD test at *α* = 0 *.* 05 using statistical software Minitab 16.0 ( www.minitab.com ) All data were tested for normality before analysis. A dendrogram of nutritional indices of examined *H. armigera* larval instars (fourth, fifth, and sixth instars) on different bean cultivars was created after cluster analysis by Ward’s method using SPSS 16.0 statistical software (IBM, www.ibm.com ).

## Results

### Nutritional indices of *H. armigera*

The results of the nutritional indices calculations of fourth instar, fifth instar, sixth instar, and those three instars combined of *H. armigera* are shown in Tables 1–4. Nutritional indices of fourth instar larvae of *H. armigera* were significantly different on various bean cultivars ( Table 1 ). The highest and lowest values of CI ( *F* = 69.51; df = 6; 197; *P* < 0.01) came from feeding *H. armigera* white kidney bean Daneshkadeh and red kidney bean Naz, respectively. The highest and lowest AD values ( *F* = 5.17; df = 6, 169; *P* < 0.01) came from feeding the fourth instar *H. armigera* larvae common bean Talash and red kidney bean Naz , respectively. The larvae fed red kidney bean Naz also showed the highest values of ECI ( *F* = 32.41; df = 6, 201; *P* <0.01) and ECD ( *F* = 31.28; df = 6, 202; *P* < 0.01). The lowest values of these indices came from *H. armigera* fed white kidney bean Daneshkadeh. Our results showed that the highest values of RCR ( *F* = 3.04; df = 6, 189; *P* <0.01) and RGR ( *F* = 34.59; df = 6, 174; *P* < 0.01) came from feeding *H. armigera* red kidney beans Akhtar and Naz, respectively. The lowest values of RCR and RGR were from feeding *H. armigera* white kidney beans Shokufa and Daneshkadeh, respectively.

**Table 1.**
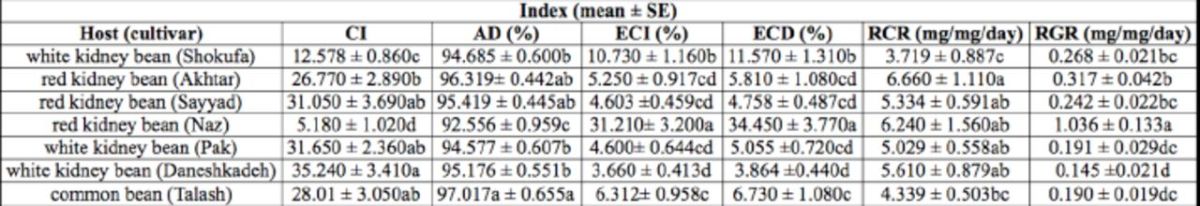
Nutritional indices of fourth instar *Helicoverpa armigera* larvae reared on seven tested bean cultivars.

The means followed by different letters in the same columns are significantly different (LSD, *P* <0.01). CI = consumption index, AD = approximate digestibility, ECI = efficiency of conversion of ingested food, ECD = efficiency of conversion of digested food, RCR = relative consumption rate, RGR = relative growth rate

The nutritional indices of fifth instar larvae are shown in Table 2 . The highest and lowest CI values ( *F* = 239.10; df = 6, 194; *P* < 0.01) came from feeding *H. armigera* fifth instar larvae white kidney beans Pak and Shokufa, respectively. The cultivars red kidney bean Akhtar and white kidney bean Daneshkadeh showed the highest and lowest values of AD ( *F* = 14.60; df = 6, 188; *P* < 0.01), respectively. According to the results, the highest and lowest values of ECI ( *F* = 3.95; df = 6, 198; *P* <0.01) were from fifth instar *H. armigera* fed red kidney beans Akhtar and Naz, respectively. The data in Table 2 shows no significant differences in ECD values of fifth instar larvae reared on various bean cultivars. The highest values of RCR ( *F* = 2.79; df = 6, 196; *P* < 0.05) and RGR (F = 3.23; df = 6, 199; *P* < 0.01) came from feeding *H. armigera* red kidney beans Sayyad and Akhtar, respectively. The lowest values of RCR and RGR were from feeding *H. armigera* white kidney bean Shokufa ( *F* = 2.79; df = 6, 196; *P* < 0.05) and red kidney bean Naz ( *F* = 3.23; df = 6, 199; *P* < 0.01), respectively.

**Table 2.**
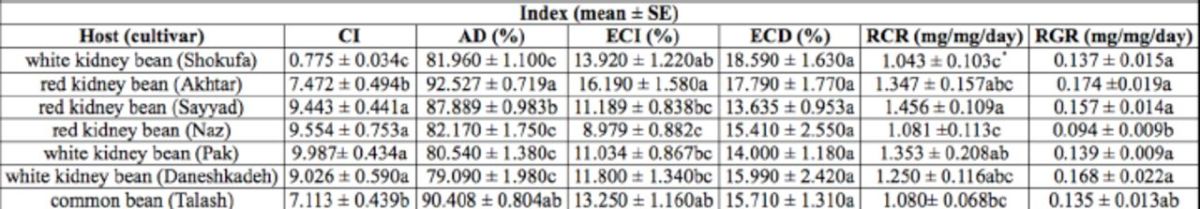
Nutritional indices of fifth instar *Helicoverpa armigera* larvae reared on seven tested bean cultivars.

The means followed by different letters in the same columns are significantly different (LSD, *P* <0.01 and *P* <0.05*). CI = consumption index, AD = approximate digestibility, ECI = efficiency of conversion of ingested food, ECD = efficiency of conversion of digested food, RCR = relative consumption rate, RGR = relative growth rate

The nutritional indices of sixth instar larvae are shown in Table 3 . The results indicated that the highest and lowest values of CI ( *F* = 38.19; df = 6, 187; *P* < 0.01) came from feeding them red kidney beans Naz and Akhtar, respectively. The larvae fed white kidney beans Shokufa and Daneshkadeh showed the highest and lowest values of AD, respectively (F = 7.26; df = 6, 180; *P* < 0.01). The highest and lowest ECI values ( *F* = 21.36; df = 6, 184; *P* <0 .01) of sixth instar *H. armigera* came from feeding them red kidney bean Akhtar and white kidney bean Pak, respectively. The ECD value ( *F* = 10.49; df = 6, 185; *P* < 0.01) of *H. armigera* sixth instars was highest when feeding them white kidney bean Daneshkadeh and lowest when feeding them red kidney bean Naz. The highest values of RCR ( *F* = 7.35; df = 6, 191; *P* < 0.01) and RGR ( *F* = 2.84; df = 6, 181; *P* < 0.05) came from feeding them red kidney bean Sayyad and white kidney bean Daneshkadeh, respectively. The lowest value of RCR came from the larvae reared on red kidney bean Akhtar, and the lowest value of RGR came from the larvae reared on white kidney bean Shokufa and red kidney bean Naz.

**Table 3.**
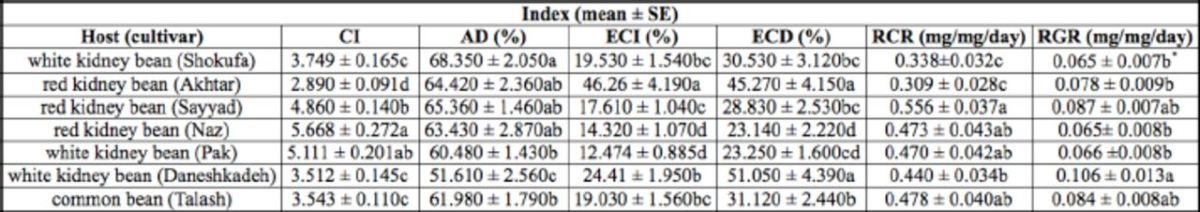
Nutritional indices of sixth instar *Helicoverpa armigera* larvae reared on seven tested bean cultivars.

The means followed by different letters in the same columns are significantly different (LSD, *P* <0.01 and *P* <0.05*). CI = consumption index, AD = approximate digestibility, ECI = efficiency of conversion of ingested food, ECD = efficiency of conversion of digested food, RCR = relative consumption rate, RGR = relative growth rate

The nutritional indices of fourth, fifth, and sixth instars combined are shown in Table 4 . The highest and lowest values of CI ( *F* = 23.23; df = 6; 159; *P* < 0.01) resulted from feeding them white kidney bean Daneshkadeh and red kidney bean Naz, respectively. The highest and lowest values of AD ( *F* = 7.34; df = 6; 173; *P* < 0.01) came from those reared on red kidney bean Akhtar and white kidney bean Daneshkadeh, respectively. The highest ECI ( *F* = 26.74; df = 6; 171; *P* < 0.01) and ECD (F = 10.57; df = 6; 175; *P* <0 .01) values came from feeding them red kidney beans Akhtar and Naz, respectively. The lowest values of ECI and ECD came from feeding them white kidney bean Pak. The highest and lowest values of RCR ( *F* = 6.00; df = 6; 147; *P* < 0.01) came from the larvae fed red kidney bean Sayyad and white kidney bean Shokufa, respectively. The highest value of RGR ( *F* = 63.7; df = 6; 164; *P* < 0.01) was from those reared on red kidney bean Sayyad, and the lowest value was from those reared on white kidney bean Pak.

**Table 4.**
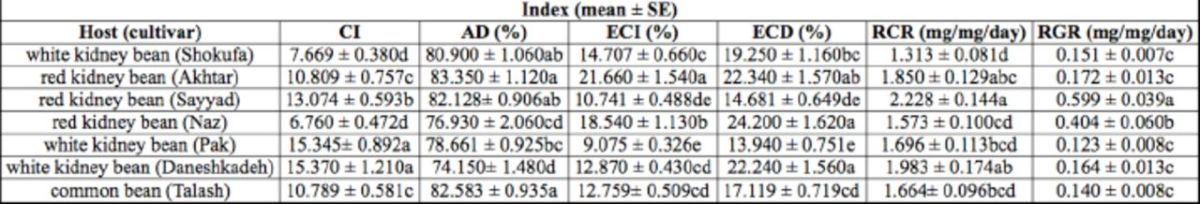
Nutritional indices of *Helicoverpa armigera* fourth, fifth, and sixth larval instars combined reared on seven tested bean cultivars.

The means followed by different letters in the same columns are significantly different (LSD, *P* <0.01). CI = consumption index, AD = approximate digestibility, ECI = efficiency of conversion of ingested food, ECD = efficiency of conversion of digested food, RCR = relative consumption rate, RGR = relative growth rate

### Cluster analysis

**Figure 1.**
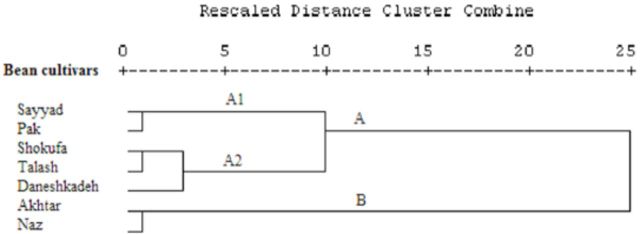
Dendrogram of various bean cultivars according to nutritional indices of *Helicoverpa armigera* in response to feeding on various cultivars (Ward’s method). High quality figures are available online.

Figure 1 shows the dendrogram resulting from cluster analysis of nutritional indices from fourth to sixth instar *H. armigera* fed various bean cultivars. The dendrogram indicates two clusters, labelled A and B. The tested cultivars were grouped within each cluster based on the comparative nutritional indices of *H. armigera* that fed on them. Cluster A consists of two subclusters, A1 (red kidney bean Sayyad and white kidney bean Pak) and A2 (white kidney beans Shokufa and Daneshkade and common bean Talash). Cluster B consists of red kidney beans Akhtar and Naz.

### Larval mortality and growth index

Table 5 shows the larval mortality rate and the growth indices. The highest and lowest percentage mortalities of *H. armigera* were in the larvae reared on red kidney bean Sayyad and common bean Talash, respectively. The results also show that the highest and lowest respective values of larval growth index came from larvae reared on common bean Talash and red kidney bean Sayyad. However, the highest value of standardized insect-growth index ( *F* = 8.31; df = 6, 168; *P* <0.01) was detected in larvae raised on white kidney bean Pak, and the lowest value was from those raised on red kidney bean Sayyad. The heaviest and lightest observed pupal weights ( *F* = 6.58; df = 6, 170; *P* < 0.01) came from larvae reared on common bean Talash and red kidney bean Sayyad, respectively.

**Table 5.**
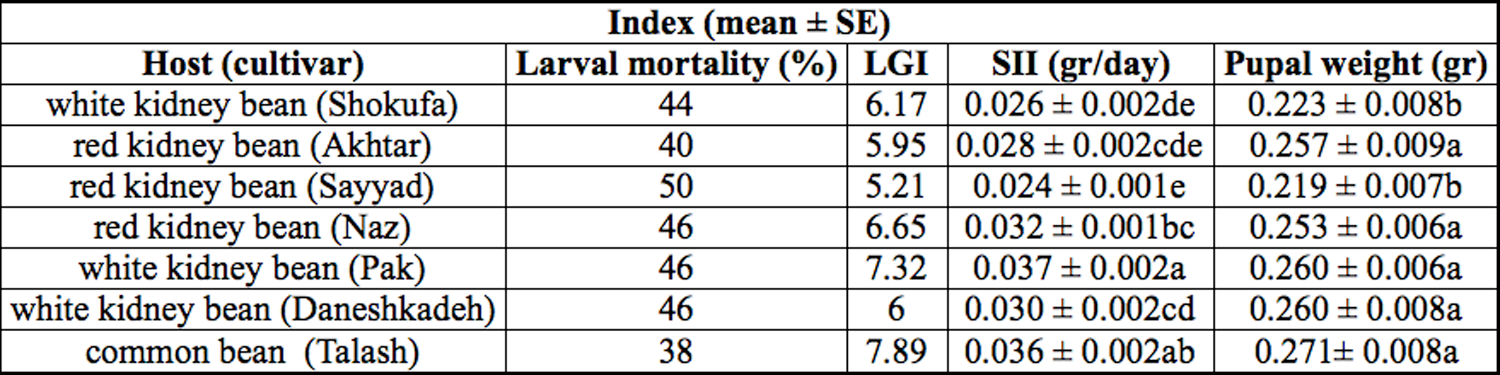
Mortality rate (%), larval growth index (LGI), standardized insect-growth index (SII), and pupal weight (g) of *Helicoverpa armigera* reared on seven tested bean cultivars.

The means followed by different letters in the same columns are significantly different (LSD, *P* <0.01)

### Protein quantification of larvae

Table 6 shows the mean protein content in the midguts of larvae. Protein contents of the midguts of fifth ( *F* = 8.06; df = 6, 14; *P* < 0.01) and sixth ( *F* = 2.87; df = 14; *P* < 0.05) instar larvae of *H. armigera* were significantly different when they were fed the various tested bean cultivars. The highest and lowest respective protein contents of fifth instar larvae came from feeding them red kidney beans Sayyad and Akhtar. The highest and lowest respective protein contents of the sixth instar larvae came from feeding them white kidney bean Pak and red kidney bean Akhtar.

**Table 6.**
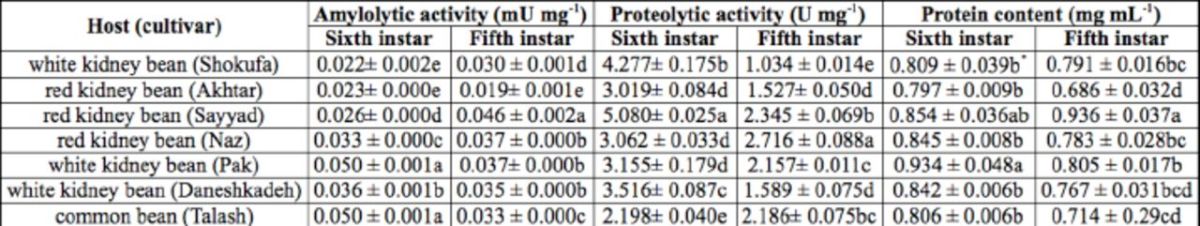
The mean (± SE) protein content (mg mL ^1^ ), proteolytic (U mg-1), and amylolytic (mU mg-1) activities of midgut extracts from fifth and sixth instar *Helicoverpa armigera* larvae fed seven tested bean cultivars.

The means followed by different letters in the same column are significantly different (LSD, *P* <0.01 and *P* <0.05*)

### General proteolytic activity of *H. armigera*

The general proteolytic activity data ( *P* < 0.01) from midgut extracts of *H. armigera* fifth and sixth instar larvae reared on various bean cultivars (for 24 hr feeding) are indicated in Table 6 . The highest proteolytic activity of fifth instar *H. armigera* was in those reared on red kidney bean Naz ( *F* = 99.80; df = 6, 14; *P* < 0.01), whereas the lowest activity was observed in the larvae fed white kidney bean Shokufa. The highest proteolytic activity of sixth instar larvae was in those reared on red kidney bean Sayyad ( *F* = 77.24; df = 6, 14; *P* < 0.01), whereas the lowest activity was in the larvae reared on common bean Talash.

### Amylolytic activity of *H. armigera*

Table 6 shows amylolytic activity ( *P* < 0.01) from midgut extracts of *H. armigera* fifth and sixth instar larvae reared on various bean cultivars (for 24 hr feeding). The fifth instar larvae reared on red kidney bean Sayyad ( *F* = 73.12; df = 6, 14; *P* < 0.01) showed the highest levels of amylolytic activity, whereas the lowest activity was observed in the larvae reared on cultivar Akhtar. The highest amylolytic activity in sixth instar *H. armigera* was observed when they were reared on white kidney bean Pak ( *F* = 358.96; df = 6, 14; *P* < 0.01) and common bean Talash, whereas the lowest activity was in the larvae reared on white kidney bean Shokufa.

### Protein and starch determination of bean cultivars given to larvae

Statistical tests indicated significant differences in protein and starch contents among the various tested bean cultivars ( *P* < 0.01; Table 7 ). White kidney bean Daneshkadeh had the highest protein content ( *F* = 5.30; df = 6, 14; *P* < 0.01), and common bean Talash had the lowest protein content. The highest and lowest respective starch contents were observed in red kidney bean Akhtar ( *F* = 34.81; df = 6, 14; *P* < 0.01) and white kidney bean Pak.

**Table 7.**
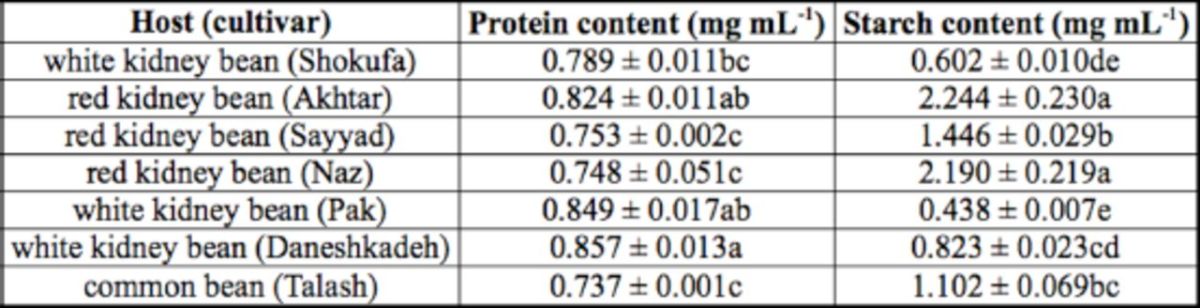
The mean (± SE) protein and starch contents (mg mL ^1^ ) of seven tested bean cultivars used for feeding *Helicoverpa armigera.*

The means followed by different letters in the same column are significantly different (LSD, *P* <0.01).

## Discussion

The suitability of a plant for hosting herbivorous insects depends on the plant’s physical and chemical aspects as well as the physiological characteristics of the phytophagous insect ( Foss and Rieske 2003 ). It is generally accepted that a set of morphological and physiological aspects, as well as secondary substances or allelochemicals of host plants, can influence plant resistance to pests. Thus, producing cultivars resistant to *H. armigera* can be one of the most basic and useful approaches incorporated into integrated pest management programs for minimizing agricultural losses caused by this pest ( Jallow et al. 2004 ).

Previous research shows that various bean cultivars have significant effects on the nutritional performance and digestive enzyme activity of *H. armigera* . ECI is a measure of an insect’s ability to incorporate ingested food into growth, and ECD, as a parallel parameter, indicates the proportion of digested food converted into net insect biomass ( Nathan et al. 2005 ). The significant differences obtained for these nutritional indices of fourth instar to sixth instar *H. armigera* larvae indicate that the tested cultivars had diverse nutritional values.

Similar to other studies ( Naseri et al. 2010a ; Arghand et al. 2011 ), we found that the nutritional responses for the fourth to sixth instars of *H. armigera* were different from each other. Insect growth is directly correlated with nutrient input; lepidopteran larvae fed high-nutrient food display faster growth rates than those fed nutrient-poor food ( Hwang et al. 2008 ). Fourth instar larvae reared on common bean Talash had the highest values of AD and some of the lowest values of RGR. The decreased growth could be due to decreased consumption, utilization, or both. It appears that the high AD could not compensate for the low ECD, which consequently resulted in growth retardation, a common response in phytophagous insects changing to a new host plant ( Lazarevic and Peric-Mataruga 2003 ). Another explanation may be increased instar duration, where large amounts of ingested food must be allotted to maintenance metabolism ( Lazarevic and Peric-Mataruga 2003 ).

In Lepidoptera, differences in digestive enzyme activity can influence nutritional responses, especially efficiency of conversion of ingested and digested food to body matter in penultimate and ultimate instars ( Patankar et al. 2001 ; Lazarevic et al. 2004 ). High ECI and ECD values in the last (sixth) instar in this study indicate greater efficiency of the conversion of ingested and digested food to biomass in this instar than in the penultimate instar. The nutritional requirements of an insect are associated with biomass and the duration of immature stages. As the amount of food ingested decreases, the insect becomes smaller and lighter due to developmental retardation ( Lazarevic and Peric-Mataruga 2003 ). Older lepidopteran larvae may digest their food less completely than younger larvae, even though their digestive capabilities are more fully developed for growth ( Dhillon and Sharma 2004 ). Lepidopteran final instar larvae may have greater needs for protein than penultimate instars, as indicated by the midguts of *H. armigera* instars fed various bean cultivars having the highest protein contents in this study. It was observed that ECD values from fourth to sixth instar larvae fed various bean cultivars increased, whereas AD values decreased.

Among the various tested bean cultivars, the highest CI values of fourth, fifth, and sixth instar *H. armigera* larvae combined came from rearing them on white kidney beans Daneshkadeh and Pak, and the rate of food intake relative to mean larval weight was highest throughout the feeding period when larvae were fed these cultivars. The CI value is approximately four-fold higher than the CI value reported by Hemati et al. (2012b) for *H. armigera* reared on common bean Khomein (3.820 ± 0.127), indicating that white kidney beans Daneshkadeh and Pak have lower nutrient values than common bean Khomein. Although *H. armigera* larvae fed white kidney bean Pak had high CI values, feeding them this cultivar also yielded the lowest values of ECI and ECD, suggesting it was more difficult to convert ingested and digested food from this cultivar into net insect biomass. Batista Pereira et al. (2002) argued that the degree of food utilization depends on the digestibility of the food source and the efficiency with which digested food is converted into biomass. Although the AD values of sixth instar *H. armigera* reared on red kidney bean Sayyad are similar to the findings of Arghand et al. (2011) rearing them on maize hybrid SC704 (66.840 ± 2.980%), the ECI and ECD values of sixth instars reared on red kidney bean Sayyad were nearly 2.5-fold higher in this study. The mean AD value of whole larval instars reared on various tested bean cultivars varied from 74.150 ± 1.480 to 83.350 ± 1.120%, which are both higher than the value reported by Cohen and Patana (1984) on bean ( *P. vulgaris)* (61.6%). Fourth, fifth, and sixth *H. armigera* instars combined reared on red kidney bean Akhtar had the highest value of AD ( Table 4 ), suggesting that it had higher nitrogen content. This value is similar to the AD value reported by Naseri et al. (2010a) rearing *H. armigera* on soybean (cultivar M9; 85.8 ± 0.064 %) and Hemati et al. (2012b) rearing them on white kidney bean Dehghan (85.308 ± 0.008%).

The ECI and ECD values of fourth instar *H. armigera* larvae reared on white kidney bean Shokufa in this study are similar to the ECI and ECD values reported by Hemati et al. (2012b) for *H. armigera* fourth instars reared on common bean Khomein (10.210 ± 0.011 and 11.580 ± 0.012%, respectively), indicating white kidney bean Shokufa and common bean Khomein are likely to have similar nutritive values. The ECI values of fifth instar *H. armigera* larvae varied from 11.034 ± 0.867 to 16.190 ± 1.580% from being fed the seven bean cultivars in this study. However, Naseri et al. (2010a) found that ECI values of fifth instar *H. armigera* larvae reared on various soybean varieties ranged from 16.300 ± 1.900% to 23.100 ± 1.400%. Therefore, soybean varieties are more nutritious hosts for the growth and development of fifth instar *H. armigera* than the seven bean cultivars tested in this study. The ECD values of fifth instar *H. armigera* larvae were not significantly different between the seven tested bean cultivars, which is in agreement with the findings of Naseri et al. (2010a) rearing them on soybean varieties. The ECI and ECD values for the combined larval instars in this study were highest when rearing them on red kidney beans Akhtar and Naz, respectively, indicating that red kidney bean Akhtar is utilized more efficiently by the larvae than the other cultivars and that the larvae fed on red kidney bean Naz were more efficient at converting digested food to body biomass, resulting in increasing larval weight. The lowest ECI and ECD values were observed when larvae were reared on white kidney bean Pak and red kidney bean Sayyad, suggesting that the larvae that fed on these bean cultivars were less efficient at converting ingested and digested food to body biomass. The efficiency of food conversion is often correlated with food utilization ( Batista Pereira 2002 ; Abdel-Rahman and Al-Mozini 2007 ). The mean ECD values of the combined larval instars that fed on the seven tested bean cultivars ranged from 13.940 ± 0.751% to 24.200 ± 1.620%, which are lower than the ECD value reported for *H. zea* fed green bean (30.70%) by Cohen and Patana (1984) . The difference between these ECD values is due to either variation in the bean cultivars or differences in the insect species.

Grouping within each cluster in the cluster analysis in Figure 1 might be due to a high level of physiological similarity between the seven tested bean cultivars. The calculated comparative nutritional indices of *H. armigera* reared on the tested bean cultivars indicate that red kidney beans Akhtar and Naz were the most suitable for *H. armigera.* White kidney bean Pak and red kidney bean Sayyad were the most unsuitable bean cultivars for this pest. White kidney beans Shokufa and Daneshkade, as well as common bean Talash, had intermediate suitability. Variations in the nutritional indices of *H. armigera* fed various bean cultivars are likely due to differences in host plant quality.

The lowest and highest respective larval mortality and larval growth index were observed in *H. armigera* reared on common bean Talash and red kidney bean Sayyad. According to Arghand et al. (2011) , larval mortality among various maize hybrids varied from 58% to 62%, and the highest and lowest values of larval growth index were observed in the larvae fed on maize hybrids SC500 (2.80) and DC370 (1.54). Comparing the results by Arghand et al. (2011) to the results of this study suggests that bean cultivars are more suitable host plants than maize hybrids for *H. armigera* development. Body weight is one of the most important biological indices of insect population dynamics ( Liu et al. 2004 ). The larvae of *H. armigera* fed red kidney bean Sayyad had the lightest pupal weights of all the larvae fed on tested host cultivars, suggesting that cultivar Sayyad was the most unsuitable host plant of those tested for *H. armigera* larvae. The pupal weight of *H. armigera* fed red kidney bean Sayyad was similar to the pupal weight reported by Naseri et al. (2010a) for *H. armigera* fed on soybean cultivar Sahar (0.216 ± 0.009 g).

The activity of digestive enzymes, including proteases and a-amylases, depends on the nature of food sources or ingested chemical compounds and enzyme-inhibitors ( Mendiola-Olaya et al. 2000 ). Insects respond to plant enzyme-inhibitors in diverse ways, such as producing inhibitor-insensitive, inhibitor-resistant, and/or inhibitor declining enzymes in their midgut ( Patankar et al. 2001 ; Volpicella et al. 2003; Telang et al. 2003 ). Since polyphagous insects have been reported to be more adaptive to different groups of inhibitors, there may be complex responses in the digestive enzymes secreted by *H. armigera* to different host plants cultivars ( Brito et al. 2001 ; Kotkar et al. 2009 ).

Variations in the protein levels of host plants can also lead to differences in the proteolytic activity of *H. armigera* that feed on them. The highest proteolytic activity value in our study was observed in fifth instar *H. armigera* larvae fed on red kidney bean Naz, but the value is lower than proteolytic activity values reported by both Naseri et al. (2010b) for *H. armigera* fed a cowpea-based artificial diet and by Hemati et al. (2012a) for *H. armigera* reared on white kidney bean Dehghan. Kotkar et al. (2009) found that legumes such as chickpea, pigeon pea, and pea have high protein content. Additionally, Patankar et al. (2001) reported that *H. armigera* larvae fed chickpea displayed more than a 2.5-3-fold higher protease activity compared to *H. armigera* larvae fed on other host plants. Higher protease activity in larvae fed chickpea, soybean, and white kidney bean Sayyad may be due to either the high protein content of the food source or the response of the insect to the ingested protease inhibitors. Although the highest general proteolytic activity of the fifth and sixth instars was observed in the larvae reared on red kidney beans Naz and Sayyad, the amount of protein in these cultivars was lower than the others. Additionally, the highest larval mortality and the lowest values of growth index, standardized insect-growth in-Journal of Insect Science | http://www.insectscience.org dex, and pupal weight were observed in those reared on red kidney bean Sayyad, indicating the presence of some protease inhibitors in this cultivar that may result in hyperproduc-tion of proteases by the midgut cells of *H. armigera.*

The highest amylolytic activity in fifth instar *H. armigera* was observed when they were reared on red kidney bean Sayyad. The highest amylolytic activity of sixth instar *H. armigera* was observed when they were reared on common bean Talash and white kidney bean Pak. The lowest amylolytic activity of fifth and sixth instars larvae was detected in larvae reared on red kidney bean Akhtar and white kidney bean Shokufa, respectively. The amylolytic activity of *H. armigera* fifth instar larvae fed red kidney bean Sayyad is approximately 7-fold lower than the amylolytic activity reported for *H. armigera* reared on white kidney bean Dehghan by Hemati et al. (2012a) . Based on the relationships between midgut protease and amylase activities, as well as the protein and starch contents of the seven tested bean cultivars, it seems that there is an insect mechanism to accurately detect and quantify the food contents and regulate the levels of these essential digestive enzymes ( Kotkar et al. 2009 ). Typically, higher protease and amylase activities in *H. armigera* larvae may be due to differences in either protein and starch contents of the diet or to the response of the insect to dietary enzyme-inhibitors.

It has been reported that the efficiency of conversion of digested food into larval biomass depends on the activity of digestive enzymes ( Lazarevic et al. 2004 ). *H. armigera* conversion efficiency was correlated with digestive enzyme activity in this study. It is possible that enzyme-inhibiting components decrease the conversion rate of food in *H. armigera* larvae reared on red kidney bean Sayyad. Based on the dendrogram, red kidney bean Sayyad was less nutritious than the other cultivars tested, as shown by lower ECI and ECD values. The lowest pupal weight, larval growth, and the highest larval mortality of *H. armigera* was observed when rearing them on this cultivar. Among the seven bean cultivars tested, the longest larval period and lowest fecundity (number of eggs laid per reproduction day) were also observed in *H. armigera* reared on red kidney bean Sayyad (unpublished data), indicating that this cultivar is an unsuitable host for growth and development of the species. Based on the nutritional responses and digestive enzyme activity observed in *H. armigera* reared on red kidney bean Sayyad, red kidney bean Sayyad is a resistant cultivar to this pest. This resistance may be due to the absence of primary nutrients necessary for *H. armigera* larval development or the presence of secondary biochemicals acting as antibiotic agents.

## Conclusions

Rearing *H. armigera* larvae on red kidney bean Sayyad resulted in reduced growth rate, decreased efficiency in converting food to body tissue, and decreased pupal weight. The low values of ECI and ECD observed when rearing *H. armigera* on red kidney bean Sayyad may be due to a lack of nutritional components or the presence of secondary chemicals. It is also probable that hyperpro-duction of protease and amylase in response to ingested enzyme-inhibitors resulted in increased energy and essential amino acid consumption, leading to developmental retardation. For a better understanding of using the interaction between *H. armigera* and its host plant species to control this insect pest, further research should be dedicated to studying the demographic parameters of *H. armigera* reared on various bean cultivars under laboratory and field conditions.

## Abbreviations

AD: assimilation efficiency/approximate digestibility
BSA: bovine serum albumin
CI: consumption index
DNS: dinitrosalicylic acid
ECD: efficiency of conversion of digested food
ECI: efficiency of conversion of ingested food
RCR: relative consumption rate
RGR: relative growth rate
